# Reducing stigma in healthcare and law enforcement: a novel approach to service provision for street level sex workers

**DOI:** 10.1186/s12939-015-0156-0

**Published:** 2015-04-09

**Authors:** Kate Bodkin, Alannah Delahunty-Pike, Tim O’Shea

**Affiliations:** Faculty of Health Sciences, McMaster University, Hamilton, Canada; Department of Family Practice, Faculty of Medicine, University of British Columbia, Vancouver, Canada

**Keywords:** Sex worker health, Women’s health, Harm reduction, Addiction

## Abstract

**Introduction:**

Providing services for street level sex workers requires a multidisciplinary approach, addressing both health and safety concerns typical of their age and gender and those that arise specific to their line of work. Despite being a diverse population, studies have identified some specific health needs for sex workers including addictions treatment, mental health. Additionally, studies have shown a higher risk of physical and sexual assault for this population. The Persons at Risk program (PAR) in London, Ontario, Canada was started in 2005 to address the specific needs of street level sex workers by using a harm-reduction model for policing and healthcare provision. This qualitative study evaluated this model of care in terms of improving access to healthcare and essential police services for street level sex workers.

**Methods:**

A total of 14 semi-structured interviews were conducted with current and former female street level sex workers enrolled in the PAR program. In addition, 3 semi-structured interviews were conducted with health and law enforcement professionals. The research team then analyzed and coded the transcripts using qualitative description to identify key themes in the data.

**Results:**

Results indicated that participants represent a vulnerable population with increased safety concerns and healthcare needs relating to addictions, mental health and infectious disease. Despite this, participants reported avoiding healthcare workers and police officers in the past because of fear of stigma or repercussions. All participants identified the harm reduction approach of the PAR program as being essential to their continued engagement with the program. Other important aspects included flexible hours, the location of the clinic, streamlined access to mental health and addictions treatment and the female gender of the police and healthcare worker.

**Conclusions:**

The PAR program provides sex workers access to much needed primary healthcare that is flexible and without judgment. In addition, they are provided with a direct avenue to access law enforcement. We feel a similar model of care could be applicable to many cities across Canada.

## Introduction

Providing services for street level sex workers requires a multidisciplinary approach, one which addresses health and safety concerns typical of a sex worker’s age and gender as well as concerns specific to their line of work. Despite being a diverse population, studies have identified specific health needs for sex workers including addictions treatment, mental health, and higher risk of physical and sexual assault [1-3].

Despite increased health needs, sex workers experience barriers to accessing health services, especially fear of stigmatization [3-6]. Furthermore, even when sex workers have primary healthcare providers, they often do not disclose their line of work [3,6-8]. A cross-sectional survey found that non-disclosure of sex work likely contributes to poor health, even when sex workers have frequent contact with healthcare professionals [7]. The following interventions to improve access to healthcare have been identified by sex workers: healthcare facilities near place of work, focusing on reproductive health and assistance with substance abuse, evening and night hours and a healthcare provider who “knows the score” [7: pg. 516].

In addition to their specific health needs, sex workers experience a myriad of safety concerns. A recent Vancouver based study highlighted the risk of violence faced by female sex workers. During an 18-month period, over half of the women experienced violence, with a quarter reporting rape [9]. Numerous other studies have found that street level sex workers often struggle with unstable housing, addictions and mental health issues [1,2]. These factors regularly intersect and expose street level sex workers to an increased risk of personal harm combined with a decreased ability to mitigate risk through self-advocacy. For example, female injection drug users, many of them sex workers, have a forty-seven times increased risk of mortality in comparison to the general female population, with the number one cause of death being homicide [10].

Street level sex workers have frequent contact with police officers. Although sex work is technically legal in Canada, most activities surrounding sex work, such as communicating in public spaces regarding sexual transactions and living off the avails of prostitution, have traditionally been illegal [11]. Canada’s prostitution laws were recently challenged in the Supreme Court of Canada (*Canada vs. Bedford*) on the basis of being found unconstitutional [12]. In response the Government of Canada recently tabled a proposed new legal framework which would criminalize purchasing sexual services and prohibit discussing the sale of sexual services in certain locations. As well prohibitions would be put in place with respect to advertising the sale of sexual services. Although little is known about the relationship between street level sex workers and police in Ontario, a Human Rights Watch report in Vancouver documented police using excessive force, arbitrary arrests and unlawful search and seizure of street involved injection drug users [13]. A qualitative study that followed this report indicated negative interactions between police and street level sex workers, leaving female sex workers feeling disempowered and distrustful of the police [14].

This paper seeks to highlight the effectiveness of a collaboration between a dedicated family physician and police officer in accessing the street level sex worker population in London, Ontario, Canada. This model of care is unique in that it bridges a gap between law enforcement and healthcare provision with a vulnerable population and plays a significant role in harm reduction. As this is a unique approach, dissemination of knowledge about the model of care, including recommendations for implementation in other urban environments is an important contribution to harm reduction and public health.

### The persons at risk program

A novel initiative called the Persons at Risk program (PAR) in London, Ontario, Canada was started to address the specific needs of street level sex workers. In 2005, the London Police Service (LPS) assigned an officer, Sergeant Lorna Bruce, to make contact with women who engaged in sex work in London. This was in response to the Robert Pickton case in Vancouver, British Columbia, where over 69 women, many of them sex workers, went missing over a period of approximately 20 years before an investigation was initiated by police. This case encouraged the LPS to reflect on their lack of knowledge regarding the safety of sex workers in London.

The PAR program initially began as a ‘tracking’ service offered to women. Sgt. Bruce would collect their DNA with the understanding it was only to be used if they went missing. However, the program has evolved significantly over the succeeding 9 years. Sgt. Bruce offers the women direct access to a police officer they can trust if safety issues should arise. Family members of street involved sex workers routinely contact Sgt. Bruce in order to communicate with their family member. This is a result of most of the women having lost touch with their families as they have become more street-entrenched. Sgt. Bruce facilitates contact between the women and their families if the women request it. Furthermore, Sgt. Bruce has made multiple community links and is able to offer the women streamlined access to addiction treatment.

In 2011, Dr. Anne Bodkin, a family physician, contacted Sgt. Bruce to establish a dedicated health program for women who engage in sex work. Since then, Dr. Bodkin and Sgt. Bruce have made contact with over 218 male and female sex workers on the streets and in jail and have provided outreach primary care as needed. Outreach occurs every Tuesday afternoon and the women are aware of the schedule. Furthermore, they have access to Sgt. Bruce via text messaging and often text her directly if they have an urgent health concern and need a house call or an escort to the hospital. As there is an element of risk associated with outreach, Dr. Bodkin reported that she would be unable to do this work without the assistance and protection of a police officer, specifically Sgt. Bruce.

Dr. Anne Bodkin has signed up approximately 90 sex workers to be part of her practice, to book appointments and drop in on an as-needed basis. Her clinic is based out of London Intercommunity Health Centre (LIHC) in London’s urban core, which provides access to a psychiatrist, social workers, housing services, HIV point of care testing, among other services.

Although there has been a long history of collaboration between police and healthcare workers in providing mental healthcare, this has rarely extended into other sectors of healthcare provision. The aim of this research was to identify the specific health and safety needs of street level sex workers in London. It also explores the past relationships that sex workers have had with healthcare services and the LPS. Finally, it tested the hypothesis that this unique collaboration between police and healthcare workers would improve access to healthcare for this vulnerable population and improve overall patient satisfaction with both the healthcare system and the police.

## Methodology

### Study design

This qualitative study comprised of 15–60 minute one-on-one semi-structured interviews with 14 women over the age of 18 who have engaged in sex work and are enrolled in the Persons at Risk program. The number of participants was left open ended during study design. Additionally, 3 interviews were held with service providers who work with this population. Voluntary informed consent was obtained prior to the interviews in a process pre-approved by the Hamilton Integrated Research Ethics Board.

### Sampling technique and inclusion criteria

The sampling technique used was snowball sampling amongst the women seen by Dr. Anne Bodkin at LIHC. At the conclusion of each interview, participants were asked if they could refer someone who fit the inclusion criteria of female, 18 and over, currently engaging in sex work or had done so in the past and accessed services at LIHC. Women below the age of 18 or who were not presently engaging in or had engaged in street level sex work in the past were excluded from this study. Service provider interviews were held with Dr. Anne Bodkin, the physician for street level sex workers at LIHC, Sgt. Lorna Bruce, the police officer who oversees the Persons at Risk program, and the London Chief of Police Brad Duncan, as he oversees the budget and renewal for the PAR program.

### Participant demographics

Participants who were currently engaging in sex work reported operating without intermediaries and met their clients on the street. Sex work was done in exchange for money or street drugs in clients’ vehicles, outdoors, in motels, or at their own residence. Demographics displayed in Table 1.

**Table 1:** **Past or present sex worker participant demographics**

**Total number of participants- 14**	
**Age**	
23-49	
**Time in sex work**	
2 months- 34 years	
**Ethnicity/race** (self-identified)	
Caucasian	11
Native	3
**Sex work status**	
Active	5
Semi-retired	1
Exited	8

### Interview process and data collection

An interview guide for past or present street level sex worker interviews was created with a set of open and closed ended questions in order to extract pertinent information, while allowing for space to explore a complex topic or narrative. Topics included demographics, time spent in sex work, healthcare experiences in hospitals and clinics, experiences with the LPS and thoughts on the collaboration between the police and a dedicated primary care physician.

Interview guides for service providers were created using the responses given during interviews held with sex workers as a guide. Topics included their experiences working with this population, barriers faced in healthcare, effectiveness of the model of care, and recommendations for implementation of this model of care in other Canadian cities.

Interviews with past or present street level sex workers took place in a closed room at LIHC. The interview with Dr. Anne Bodkin took place in a closed room at LIHC. The interview with Sgt. Lorna Bruce took place in her office at the London Police Service. The interview with the London Chief of Police Brad Duncan took place in his office at the London Police Service.

Interviews were conducted until clear, persistent themes were identified and researchers felt satisfied that the questions had been explored in depth and saturation was reached. This happened after 14 one-on-one semi-structured interviews had been conducted with street level sex workers. Participants who identified as street level sex workers now or in the past, received reimbursement for travel and a $20 gift card for a local coffee shop.

### Transcription

Interviews were tape-recorded and audio files of the tape recordings were transcribed verbatim with identifying data removed by two members of the research team. One member of the research verified all of the transcripts for accuracy.

### Coding

Based on the transcript data, the research team created a coding guide that underwent seven iterations. All members of the research team met and agreed upon the final coding guide.

### Data analysis through qualitative description

Once the coding guide was complete, two members of the research team divided the transcripts and coded according to the guide, while using a qualitative descriptive analytical approach, which aims “to describe the informant's perception and experience of the world and its phenomena” [15]. This method of analysis was used as it was found to be most appropriate for the nature of the responses and to best represent the narrative style interviews and relaying interview responses as accurately as possible in the analysis. According to Margarete Sandelowski, “[q] ualitative descriptive study is the method of choice when straight descriptions of phenomena are desired” [16]. As this was an entirely qualitative study, the research team felt that this was the most appropriate method of analysis for information and data collected throughout the study. All members of the research team agreed on the final analysis of the data.

### Ethical reflection

As most of the interviews were conducted with a vulnerable population that had a high probability of exposure to violence and residual trauma, an ethical reflection must be considered. During the informed consent process, participants were told that they could stop the interview at any time or skip over questions, if needed. They were also assured that if requested, their data could be removed from the study at any time. In addition to consent, at the conclusion of the interview, participants could be referred to a psychologist or counseling services at LIHC, if needed. Dr. Anne Bodkin, who introduced the participants to the interviewers, assessed them to ensure they had the ability to give their informed consent to participate.

## Results

### Health and safety issues

It was evident that the participants that identified as sex workers represent a vulnerable population with complex and interconnected health issues. All participants spoke of struggles with addiction to injection drugs. These addictions were inextricably linked to their line of work. This was highlighted both by the fact that many were exchanging sex for drugs directly and that the participants who had exited sex work were only able to do so once they received appropriate addictions treatment and had rehabilitated.

*“Interviewer: “How often were you doing* (sex work)*?*

*Participant: Oh sometimes everyday and sometimes up to 10 – 20 times a day, depending on how bad, you know I needed drugs. how much money I’d get at one time and if that lasted me long enough”.*

Many participants struggled with health complications directly related to their substance abuse. The majority of participants felt that drug use had a direct negative impact on their health either in the present or in the past. Many reported infectious diseases related either to sex work or to injection drug use. This included 7 participants with Hepatitis C and 2 participants with HIV. Furthermore, while all participants described mental health issues, half reported psychiatric episodes, including frank psychosis or suicide attempts that warranted hospitalization.

Participants reported frequent emergency services use. The mean number of self-reported lifetime visits to the emergency was 19.1 times with a range between 1 and >50. Reasons for accessing emergency services were fairly consistent across participants, including accidental or intentional drug overdoses, infected abscesses, endocarditis, bacteremia, and mental health issues. Many participants discussed fear of sexual and physical abuse as a part of their everyday lives, both in their interpersonal relationships and with clients. One participant described feeling scared every time she walked up to a car to initiate a transaction.

*“I’m lucky I made it this far . . . like I’ve almost been murdered a few times, I’ve had guns pulled on me, I’ve been strangled, I’ve been beaten with fists. . . you wouldn’t believe what I’ve been through and I’m still here, you know”.*

Statistics obtained from LIHC regarding their clients paint a similar picture for sex workers in London as a whole. Of the 87 sex workers currently registered with the PAR program, only 9 had their first encounter at the clinic itself. The rest were introduced to Dr. Anne Bodkin on the street, in jail, at shelters, or during a home visit. 34 clients have only accessed healthcare during outreach, and the other 53 have made their way to the clinic for healthcare. This highlights the absolute essential role that outreach work plays in the success of the PAR program. Approximately 1/3 of the clients (32/87) were not practicing ‘safe sex’ at the time of first contact. The top 10 issues addressed by Dr. Bodkin over a 10-month period are depicted in Table 2. They are strikingly similar to the health and safety issues highlighted by our in-depth interviews.

**Table 2:** **Top 10 health issues addressed**

**Issue addressed**	**Frequency (%)**
Drug abuse	20
Viral hepatitis	9.5
Housing issues	6.8
HIV/AIDs	6.5
Affective psychosis	5.4
Health maintenance/preventative medicine	4.5
Post-traumatic stress disorder	4.5
STI counseling/testing	4.2
Medication renewal/change	3.2
Assault/harmful event	2.1

Data provided by LIHC statistician, included 755 visits with 96 different health issues to Dr.Bodkin over 10 months.

### Avoidance of police and healthcare services

Despite reports of multiple health and safety issues, participants reported avoiding seeking help from healthcare workers or police officers to address their concerns. 13 of the 14 participants described negative experiences within the healthcare system by feeling judged, or ‘looked down upon’.

*“…I’ve been to hospitals where I’ve felt dirty and I’ve felt these people just look at me like, just like I’m a stupid prostitute drug addict like get out of my hospital almost you know and lots of times I wouldn’t go. I’d pick out my own abscess and they’d get re-infected and like I have scars because I picked them myself because . . . I didn’t want to go to the hospital because of the judgment. And . . . the stigma behind the addiction part of it”.*

Some participants felt they had received sub-par care because of their struggles with drug addiction and consequent multiple visits to the hospital. Participants described having pain medication withheld, poor treatment because they could not be trusted with having IV lines in, or having health complaints not taken seriously because all of their issues were simply ‘chalked up’ to drug abuse.

*“…they brought a security guard in, tore my room apart thinking they were gunna find drugs and they didn’t and they still kicked me out even though I wasn’t using* [IV drugs] *and my PICC line and they said the oral medications* [are] *a lot less effective but that’s what we’re gonna give you…”.*

Many participants reported avoiding seeking healthcare, both at the hospital and in a primary care setting. This would result in participants delaying addressing health issues until the situation became critical. Many of the participants lacked consistent primary care or preventative healthcare before enrolling in the PAR program. For example, one participants stated that she had never received a Pap smear or STI testing despite over 5 years of engaging in sex work and injection drug use.

Although this issue was not explored in depth, some participants reported a negative relationship with police prior to the PAR program or in other cities, particularly surrounding feeling judged or targeted. Many had been arrested or taken in by police several times due to the illegal nature of soliciting sex in the streets and/or drug use. Thus, many actively avoided police before meeting Sgt. Bruce.

### Evaluation of the PAR program

Participants consistently stated that the collaboration between a dedicated physician and police officer using a harm-reduction approach could address barriers faced by sex workers, while providing essential services for a vulnerable population.

*“It really. . . puts strength, it puts power, it puts support…gives you a little bit of safety. It’s like, you know if I’m sick I know I could go to Dr. Bodkin. If . . . that John beat me up and raped me . . .I know I could go to Lorna”.*

Positive attributes of the PAR program identified by participants, included the lack of judgment by healthcare providers or police officers, feeling ‘believed in’ or ‘worthy’ of help because a person in a position of power wanted to help them, and feeling safer because they had direct contact with a police officer they felt was ‘for them’.

*“…And (she) said to me that, I just want you know that there’s this John…He wears a baseball cap he’s driving his parents’, his dad’s truck. . . he’s raping the women. Be careful. Please be careful. And I just thought, this is weird man, you know I’ve got dope in my bra, pills over here and I’m just like this is really weird right. She didn’t check me out or nothing. So that’s when sort of the trust started to develop”.*

(Participant describing her first interaction with Sgt. Bruce).

Essential components of the program identified include flexible hours and drop-in access of both the clinic and the police officer, streamlined access to addictions and mental health treatment, being located in the part of town that their work takes place, and community outreach work by service providers. The female gender of both the police officer and physician was repeatedly identified as being very important to participants as many reported feeling uncomfortable with men, often due to a history of abuse by men.

Finally, two thirds (8/14) of the participants interviewed had successfully exited sex work and identified the PAR program as being integral to this transition. Discussion of the factors that contribute to exiting sex work is outside the scope of this study. However, participants identified an inextricable link between sex work and drug use. The PAR program offers participants immediate access to addiction treatment if they request it. Furthermore, many participants identified a feeling of self worth and increased self-esteem associated with their relationships with healthcare providers and Sgt. Bruce that may have contributed to them successfully addressing addictions.

*“…I didn’t just quit* (injection drug use) *automatically overnight kind of thing but I remember…Lorna, Dr. Bodkin, Henry* (a social worker)…*it was like if all these people believe in me then maybe there is somebody worth saving…”*

## Discussion

The results of this study indicate that street level sex workers in London, Ontario represent a vulnerable population due to increased health and safety risks. Increased healthcare needs relate specifically to addictions, sexual health, mental health and infectious disease. Furthermore, their line of work and addictions expose them to frequent sexual and physical abuse. This reinforces what is already known in the literature- that there is a commonality to the experience of being involved in street level sex work and that this line of work exposes women to high risks both from a health and safety point of view [1-3,6,17]. This can be used to inform service providers about priority areas for resource allocation to this historically marginalized population.

Before the PAR program began, street level sex workers in London reported a negative relationship with healthcare workers and the police. This resulted in avoidance of police and health services, which likely has served to reinforce negative health outcomes and increased exposure to violence. Although research has well-documented the fear of stigmatization that acts as a barrier to accessing health services [3-6], there is very little research done on the relationship between sex workers and police. This avoidance of police services is likely a phenomenon that is present throughout Canada, as we know that sex work remains at least partially criminalized and thus sex workers would fear repercussions if they engage with police. Providing sex workers an avenue to access police services is essential to ensuring their safety. This was highlighted during the study period as there was a string of violent attacks by a John on some of the sex workers. The women that were attacked texted Sgt. Bruce and she escorted them to the police station to make statements. This resulted in an arrest made within 10 days of the reported first incidence. This is a very different approach to the response of other police forces to violence against sex workers in large Canadian cities [18,19].

Finally, collaboration with law enforcement ensures the safety of healthcare providers in their outreach work and allows them to access the sex workers in their homes and on the street if they are too sick or unwilling to present to a clinic. Dr. Anne Bodkin made it clear that she would not feel safe in her outreach work without Sgt. Bruce. Study participants highlighted the outreach component as being absolutely essential to the success of the program.

A limitation of this study was the small number of participants. However, there were strikingly consistent themes between interviews and thus we feel the data is robust. Furthermore, we feel this is a unique study population and a unique model of care and thus an important avenue for study. Additionally, there was a clear overrepresentation of participants who had exited sex work. This represents a sampling bias, as participants who are no longer using drugs were probably more likely to show up to scheduled appointments. However, interviews with these participants offered a glimpse into a world that is not often studied and depicts a successful case study of addictions rehabilitation and exiting of sex work. Future research into the factors that contribute to exiting sex work and barriers faced by these sex workers is recommended.

Although there are a number of studies of the health status and barriers faced by street level sex workers in Vancouver [9,20-23] and one in Montreal [21], these have not been replicated in other cities in Canada. The collaboration featured here between a family physician and a community police officer to address the health and safety concerns of sex workers, appears to be the first of its kind in North America. Given that this model of care is the first of its kind, evaluating its effectiveness in terms of addressing these barriers is crucial if other cities are to adopt a similar model. Informal discussions with study participants who had worked in other Canadian cities and with healthcare providers across Canada indicate that street level sex workers experience similar barriers to accessing appropriate health services and police protection in other cities. Thus, the authors feel strongly that the harm reduction approach and the unique collaboration between police and healthcare providers employed by the PAR program could be replicated with success in many other cities.

## Conclusions

Street level sex workers experience a myriad of barriers to accessing much needed healthcare and police resources, including stigmatization and fear of repercussions. As a result, they report avoidance of healthcare workers and the police. The Persons at Risk program is an effective harm-reduction policing model combined with streamlined and focused healthcare provision. This program allows sex workers to access much needed primary healthcare that is flexible and without judgment. In addition to the healthcare component, sex workers are provided with an avenue to access law enforcement if the need arises. This unique program could be applied broadly in urban areas across North America in order to effectively address these barriers and respond to the high needs of this vulnerable population.
